# Efficacy and safety of anti-PD-1/PD-L1 antibodies in patients with relapsed refractory diffuse large B-cell lymphoma: A meta-analysis

**DOI:** 10.1515/biol-2025-1129

**Published:** 2025-08-05

**Authors:** Jiawen Zhang, Lei Xu, Caifeng Sun, Zonghua Huang, Ji Ma, Liang Wang

**Affiliations:** Clinical Medicine School, Shandong Second Medical University, No. 4948, Victory East Street, Weifang, Shandong, 261000, China; Department of Hematology, Shengli Oilfield Central Hospital, No. 31, Jinan Road, Dongying District, Dongying, Shandong, 257034, China; Department of Medical Imaging, Shengli Oilfield Central Hospital, No. 31, Jinan Road, Dongying District, Dongying, Shandong, 257034, China; Department of Hematology, Shandong Cancer Hospital and Institute, Shandong First Medical University and Shandong, Academy of Medical Sciences, No. 440, Jiyan Highway, Huaiyin District, Jinan, Shandong, 250117, China; Qilu Hospital of Shandong University, Dezhou Hospital, No. 1 Shangde Sixth Road, Decheng District, Dezhou, Shandong, 253000, China

**Keywords:** PD-1/PD-L1 blockade, immune checkpoint molecule, relapsed/refractory diffuse large B-cell lymphoma, treatment, meta-analysis

## Abstract

This article conducts a meta-analysis to evaluate the safety and efficacy of PD-1/PD-L1 inhibitors in patients with relapsed/refractory diffuse large B-cell lymphoma (R/R DLBCL). A total of 63 papers were initially retrieved, and eight clinical studies were collected. The estimated effect of ORR was [OR = 0.40, 95% CI 0.29–0.51; *p* = 0.08], the estimated effect of complete response rate was [OR = 0.21, 95% CI 0.14–0.31; *p* < 0.001], while the estimated effect of 1-year progression-free survival was [OR = 0.33, 95% CI 0.22–0.47; *p* = 0.01]. The estimated effect of 1-year OS was [OR = 0.67, 95% CI 0.55–0.77; *p* = 0.05]. In addition, the estimated effect of grade 3 adverse events was [OR = 0.33, 95% CI 0.22–0.46; *p* = 0.01]. Overall, PD-1/PD-L1 inhibitors demonstrated suboptimal therapeutic efficacy in the selected trials for R/R DLBCL. However, combining PD-1/PD-L1 inhibitors with CAR-T showed potential for improved treatment outcomes. Additionally, PD-1/PD-L1 inhibitors were found to be safe and well-tolerated in patients with R/R DLBCL.

## 1 Introduction

Diffuse large B-cell lymphoma (DLBCL) represents the most prevalent subtype of aggressive lymphoma, where approximately 60–65% of patients achieve remission after initial treatment. Nevertheless, the prognosis for relapsed or refractory cases continues to pose significant clinical challenges, with the optimal therapeutic strategy remaining under active investigation. While autologous stem cell transplantation (ASCT) has emerged as a cornerstone therapeutic intervention for relapsed/refractory (R/R) DLBCL in recent years, clinical evidence reveals suboptimal outcomes: Patients achieving partial response (PR) after second-line salvage chemotherapy who subsequently undergo ASCT demonstrate a strikingly limited median overall survival (OS) of 4.4 months, accompanied by 1-year and 2-year OS rates of 23 and 16%, respectively [1]. Notably, even in transplant-eligible populations, ASCT fails to achieve long-term disease-free survival rates exceeding 50%. Beyond ASCT, the therapeutic landscape for R/R DLBCL encompasses multiple emerging modalities, including chimeric antigen receptor T-cell immunotherapy (CAR-T) therapy, next-generation monoclonal antibodies, antibody–drug conjugates, bispecific antibodies, targeted small molecule inhibitors, and allogeneic stem cell transplantation. Critical questions regarding the optimal sequencing paradigms and combination strategies for these interventions persist as focal points of contemporary clinical research.

Programmed cell death protein-1 (PD-1), a pivotal immune checkpoint receptor, orchestrates peripheral tissue T cell activity while maintaining immune tolerance during infection-induced inflammatory responses. Emerging evidence highlights substantial infiltration of regulatory T cells (Tregs) in diverse tumor microenvironments (TMEs), where tumor-infiltrating lymphocytes exhibit marked PD-1 upregulation. This molecular signature promotes Treg expansion through PD-1/ligand interaction. The receptor engages two structurally distinct ligands: programmed cell death ligand 1 (PD-L1) (B7-H1/CD274) and PD-L2 (B7-DC/CD273), with PD-L1 overexpression being extensively characterized in multiple malignancies. Clinical studies have consistently demonstrated aberrant PD-L1 expression patterns in solid tumors including non-small cell lung cancer [2], melanoma [3], renal cell carcinoma [4], as well as hematological neoplasms such as relapsed/refractory classical Hodgkin lymphoma [5,6]. Of particular clinical relevance, the spatial distribution and quantitative expression of PD-1/PD-L1 within both tumor parenchyma and infiltrating immune cells have gained recognition as predictive biomarkers for patient outcomes in contemporary oncology.

Therapeutic inhibitors targeting the PD-1/PD-L1 axis abrogate the interaction between PD-1 receptors and their ligand PD-L1 expressed by activated T cells, thereby rescuing T cell exhaustion and reinvigorating antitumor immunity [7]. Pivotal clinical trials have established the clinical precedence of PD-1 inhibitors surpassing conventional therapies across malignancies, notably advanced melanoma [8], non-small cell lung cancer [9,10], and multiple myeloma [11]. Mechanistic studies reveal that anti-PD-L1 antibodies achieve precision targeting of the PD-1/PD-L1 pathway while preserving PD-1/PD-L2 signaling critical for peripheral immune homeostasis, thereby reducing immune-related toxicities [12]. Illustratively, a phase 1 trial evaluating Nivolumab in R/R DLBCL demonstrated an overall response rate of 36% and complete response (CR) rate of 18%, with median progression-free survival (PFS) limited to 7 weeks after 2-year treatment [13]. Younes et al. reported a combination regimen of Ibrutinib plus Nivolumab yielding ORR and CR rates of 36 and 16%, respectively, in R/R DLBCL [14]. Similarly, Armand et al. conducted a phase 2 study showing Pidilizumab monotherapy achieved ORR of 51% and CR of 34% in this population [15]. Notably, the existing evidence landscape lacks comprehensive systematic evaluations assessing both efficacy profiles and safety parameters of PD-1/PD-L1 inhibitors specifically in DLBCL. This study undertakes a rigorous meta-analysis to address this knowledge gap, aiming to provide evidence synthesis for optimizing clinical decision-making in this challenging patient cohort.

## 2 Materials and methods

### 2.1 Retrieval strategy

We executed a systematic search strategy across PubMed, Cochrane Library, and EMBASE databases to identify studies published from January 1, 2014 through January 1, 2024. To account for regional therapeutic variations, particularly the commercial availability of multiple PD-1 inhibitors in China, our search incorporated both generic terms (“PD-1 blockade” or “programmed death-1 blockade”) and proprietary agent nomenclature: Pembrolizumab, Nivolumab, Sintilimab, Camrelizumab, Tislelizumab, and Toripalimab. To enhance search specificity, we employed combinatorial search strings integrating these target keywords with disease-specific terms including “diffuse large B-cell lymphoma” and “refractory or relapsed (R/R)” during secondary screening of the preliminary search results.

### 2.2 Inclusion criteria

Inclusion criteria included: (1) the study population consisted of patients diagnosed with R/R DLBCL, (2) the study investigated the efficacy of PD-1/PD-L1 inhibitors in treating R/R DLBCL, (3) the article presented data on ORR, PFS, OS, and drug-related adverse events (AEs), and (4) literature included in the review was restricted to English and Chinese languages.

### 2.3 Exclusion criteria

Exclusion criteria included: (1) irrelevant articles were excluded from consideration; (2) articles lacking the specified outcome measures and data on adverse drug reactions were also excluded; (3) letters, case reports, and reviews were not included in the analysis; (4) republished articles were not included in the review; and (5) conference articles and abstracts presenting only stage summaries were excluded from the analysis.

### 2.4 Quality evaluation and data extraction

Two independent investigators performed the study selection process using predefined eligibility criteria, with screening conducted in duplicate to ensure reproducibility. Discrepancies were resolved through deliberation or adjudication by a third researcher when consensus could not be reached. Data extraction focused on capturing key parameters: patient demographics (age distribution in case and control cohorts), sample size, therapeutic outcomes (ORR, CR, OS, PFS), first author identification, and publication timeline. AEs were systematically classified into hematologic toxicities (neutropenia, thrombocytopenia, anemia) and non-hematologic events. Methodological rigor was evaluated using the validated Methodological Index for Non-Randomized Studies (MINORS) tool [16], which assesses eight critical domains: (1) explicitly defined study objective, (2) consecutive patient enrollment, (3) prospective data acquisition, (4) endpoint alignment with research aims, (5) blinded endpoint assessment, (6) clinically appropriate follow-up duration, (7) follow-up completion rate ≥95%, and (8) *a priori* sample size calculation.

### 2.5 Statistical analysis

The ORR represented the proportion of patients achieving CR and PR. All data processing was conducted utilizing RevMan 5.4 software. The combined OR and its corresponding 95% confidence interval (CI) were calculated using the formula: pf = OR/(1 + OR), lower limit of 95% CI (LL) = LLOR/(1 + LLOR), and upper limit of 95% CI (UL) = ULOR/(1 + ULOR). Heterogeneity analysis was performed using two methods: the *I* ^2^ test and the *Q* test. Heterogeneity was deemed small if *I* ^2^ was less than 50% and *Q* test had a *p*-value greater than 0.1, in which case the fixed-effect model was employed for analysis. Conversely, if there was significant heterogeneity (*I* ^2^ ≥ 50% or *Q* test *p*-value ≤0.1), the random-effects model was utilized for combined analysis.

## 3 Results

### 3.1 Study characteristics and quality assessment

**Figure 1:**
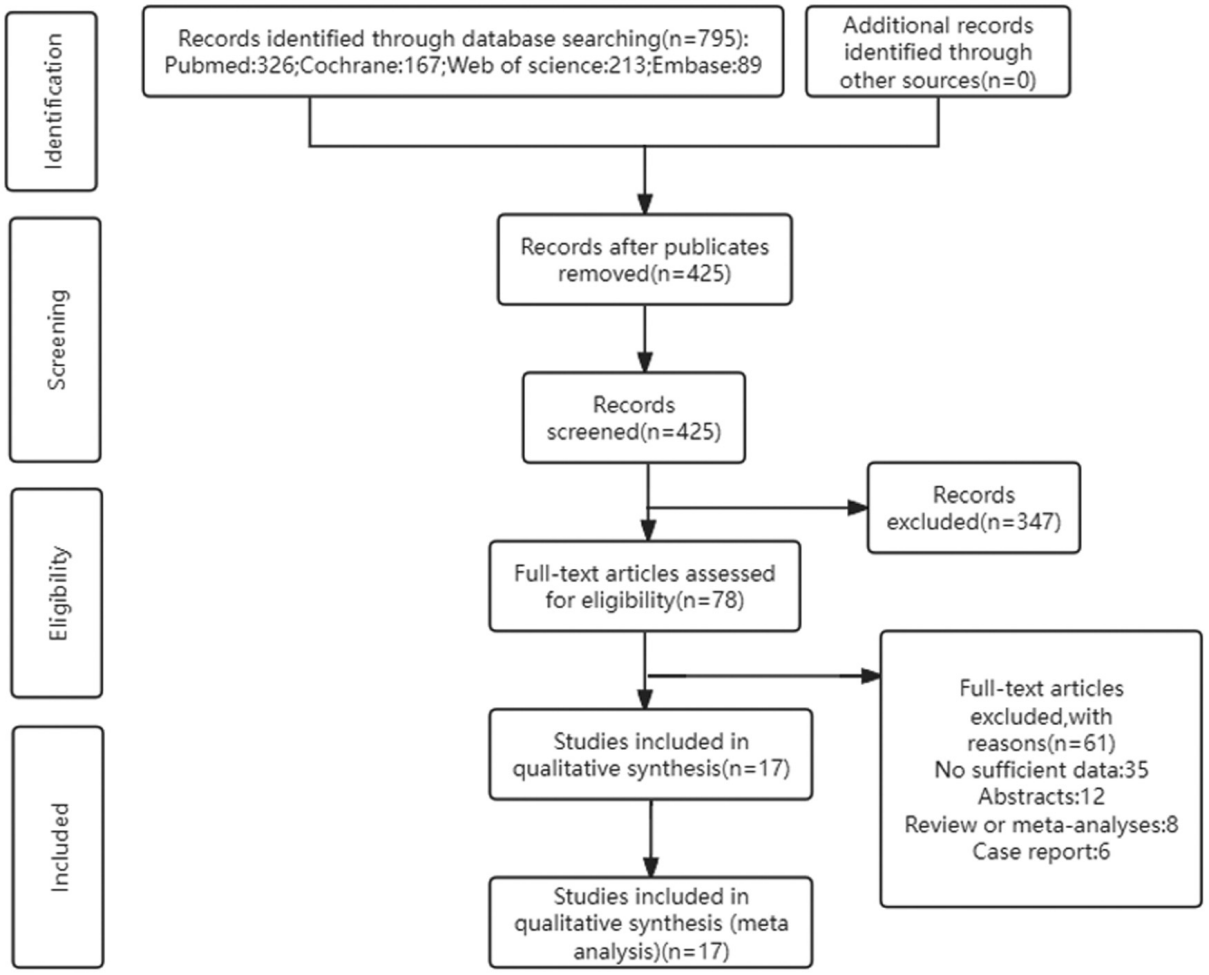
Flow chart of the literature search and study selection.

A total of 63 papers retrieved from the four databases underwent screening based on the predetermined inclusion and exclusion criteria. Ultimately, 17 clinical case–control studies [14,17–28] were selected, comprising 576 cases with R/R DLBCL. All included studies assessed the impact of anti-PD-1/PD-L1 antibodies on R/R DLBCL. The study screening process is depicted in Figure 1. Detailed information for each included study is provided in Table 1. The quality assessment of papers using the MINORS revealed moderate quality, with scores ranging from 10 to 13. The MINORS scores for the included papers are presented in Table S1.

**Table 1:** Included in the article baseline table

Author & year	Trial phase	Number	Age	Follow-up duration	Treatment	ORR (%)	CRR (%)	Adverse events (>grade 3)
Philippe Armand (2013)	2	66	57 (19–80)	16 months	Pidilizumab	51.5	34.0	Neutropenia: 19%
								Thrombocytopenia: 8%
Anas Younes (2019)	1/2a	65	65 (54–71)	18.4 months (15.6–19.4)	Ibrutinib + Nivolumab	44.6	13.8	Anemia: 26%
								Neutropenia: 20%
								Rash: 12%
Stephen M. Ansell (2018)	2	121	a: 62 (24–75) b: 68 (28–86)	9 months	Nivolumab	8.0	2.0	Neutrophils decreased: 4%
								Platelets decreased: 3%
								Lipase increased: 3%
Alexander M. Lesokin (2016)	1b	11	65 (23–74)	7 weeks (6–29)	Nivolumab	36.0	18.0	Pneumonia: 4%
								Anemia: 4%,
								Low white blood cells: 4%
Vincent Ribrag (2021)	1b	32	68 (41–87)	—	Durvalumab + Tremelimumab	6.0	0.0	—
Alex F. Herrera (2018)	1b/2	34	GCB:68 (22–82) non-GCB 67 (39–82)	17.5 months (0.2–23.6)	Ibrutinib + Durvalumab	24.0	18.0	Neutropenia: 26%
								Fatigue: 12%
								Dyspnea: 12%
Liqin Ping (2023)	—	67	54 (23–74)	24.7 months (1.4–39.6)	Sintilimab/Camrelizumab/Toripalimab/Pembrolizumab + ICE	62.7	43.3	Neutropenia: 7.5%
								Anemia: 5.0%
								Thrombocytopenia: 9.0%
Yan Qin (2021)	2	30	56.5 (20–78)	21.3 months (9.6–24.2)	Toripalimab/Pembrolizumab/Nivolumab/Sintilima + Rituximab	53.3	6.7	Interstitial pneumonia: 7%
								Hypophysitis:
								3%
A. Davies (2021)	2	41	73 (23–85)	—	Atezolizumab + R-GemOx	39.0	13.0	Thrombocytopenia: 32%
								Neutropenia: 10%
								Pneumonia: 10%
								Fever: 10%
Juan Mu (2021)	2	26	52	—	CD19 CAR-T + Sintilimab	65.39	42.31	Neutropenia: 54%
								Thrombocytopenia: 35%
								Fatigue: 31%
								Fever: 27%
								Chills: 23%
Teng Yu (2023)	1b	11	50 (40–70)	31 months (2–34)	CD19 CAR-T + Tislelizumab	72.7	45.5	CRS: 19%
Chunmeng Wang (2021)	—	5	40 (35–54)	21.8 months	After failure of CD19/20 CAR-T therapy, Sintilimab/Camrelizumab	60.0	40.0	—
Qian W. (2021)	1b	8	45.5 (38–65)	—	CD19 CAR-T + Tislelizumab	75.0	57.1	ICANS: 25%
James Godfrey (2023)	1	6	51 (21–79)	3.8 years (2.9–5.1)	Vorinostat + Pembrolizumab	33.0	17.0	Neutropenia: 17%
								Hypertension: 17%
Carmelo Carlo‐Stella (2022)	1/2	17	64 (23–75)	24 weeks	Isatuximab + Cemiplimab	5.9	5.9	Decreased appetite: 12%
								Abdominal pain: 6%
								Peripheral edema: 6%
U. Jaeger (2021)	1b	12	62 (35–79)	4 months	CD19 CAR-T + Pembrolizumab	33.3	16.7	Neutropenia: 33%
Nitin Jain (2023)	2	24	64.5 (47–88)	46.3 months (1.2–61.3)	Nivolumab + Ibrutinib	42.0	34.0	Lung infection: 4%
								Lipase: 4%
								Uveitis: 4%
								transaminase: 4%

### 3.2 Heterogeneity test and estimated effect analysis of ORR

**Figure 2:**
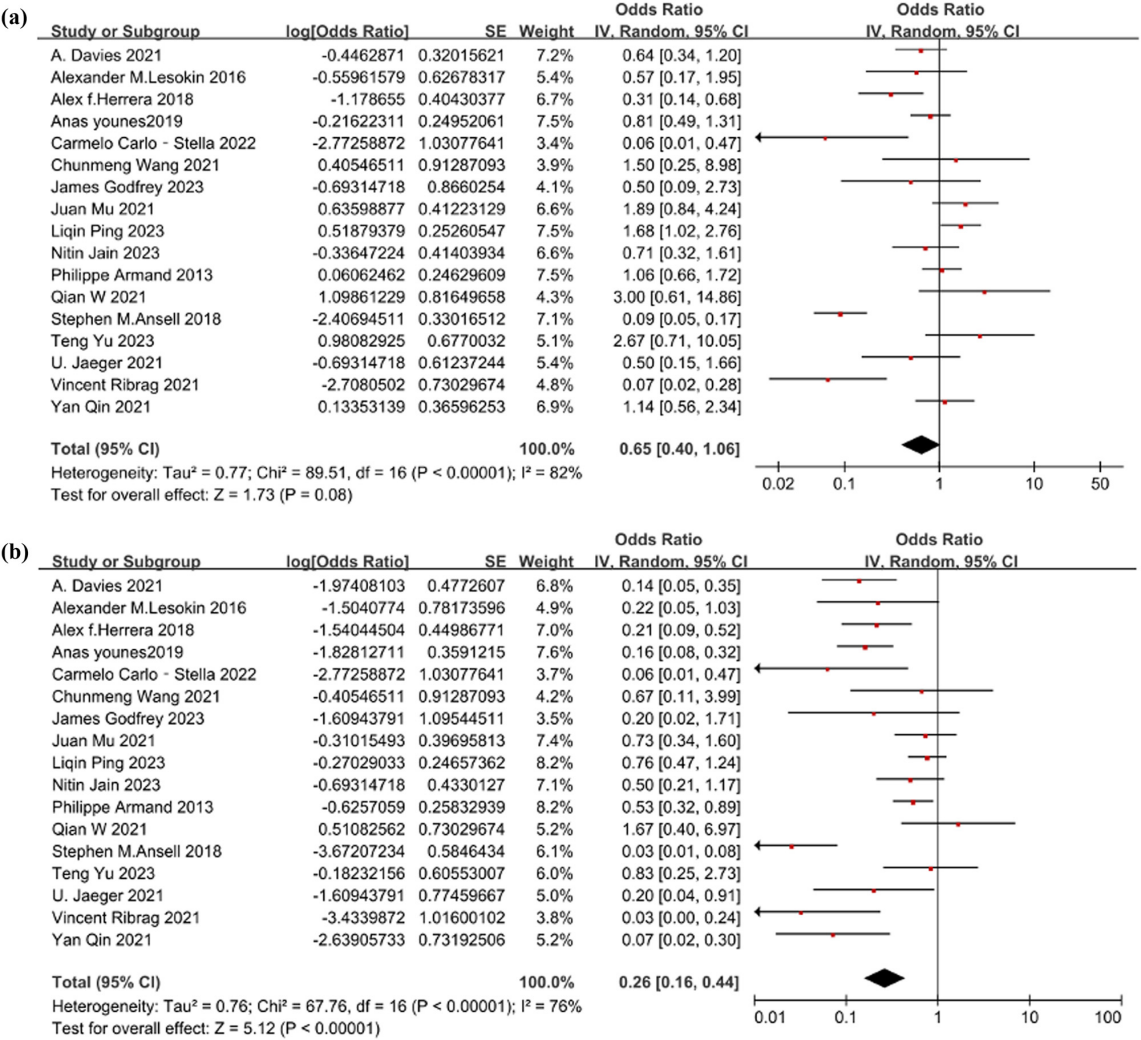
(a) The forest plot of the level of ORR. The heterogeneity test result was *Q* = 89.51 (*p* < 0.001) and *I* ^2^ = 82%. The estimated effect was [OR = 0.40, 95% CI 0.29–0.51; *p* = 0.08]. (b) The forest plot of the level of CRR. The heterogeneity test result was *Q* = 67.76 (*p* < 0.001) and *I* ^2^ = 76%. The estimated effect was [OR = 0.21, 95% CI 0.14–0.31; *p* < 0.001].

The heterogeneity test results for the level of ORR were as follows: *Q* = 89.51 (*p* < 0.001) and *I* ^2^ = 82%. These findings indicate substantial heterogeneity among the studies, warranting the use of a random-effects model for analysis. The estimated effect of ORR was [OR = 0.40, 95% CI 0.29–0.51; *p* = 0.08]. Figure 2a illustrates the forest plot depicting the level of ORR.

### 3.3 Heterogeneity test and estimated effect analysis of CRR

The heterogeneity analysis for the level of CRR yielded *Q* = 67.76 (*p* < 0.001) and *I* ^2^ = 76%, indicating significant heterogeneity among the studies. Therefore, a random-effects model was employed for analysis. The estimated effect of CRR was [OR = 0.21, 95% CI 0.14–0.31; *p* < 0.001]. Figure 2b depicts the forest plot illustrating the level of CRR.

### 3.4 Heterogeneity test and estimated effect analysis of 1-year PFS

**Figure 3:**
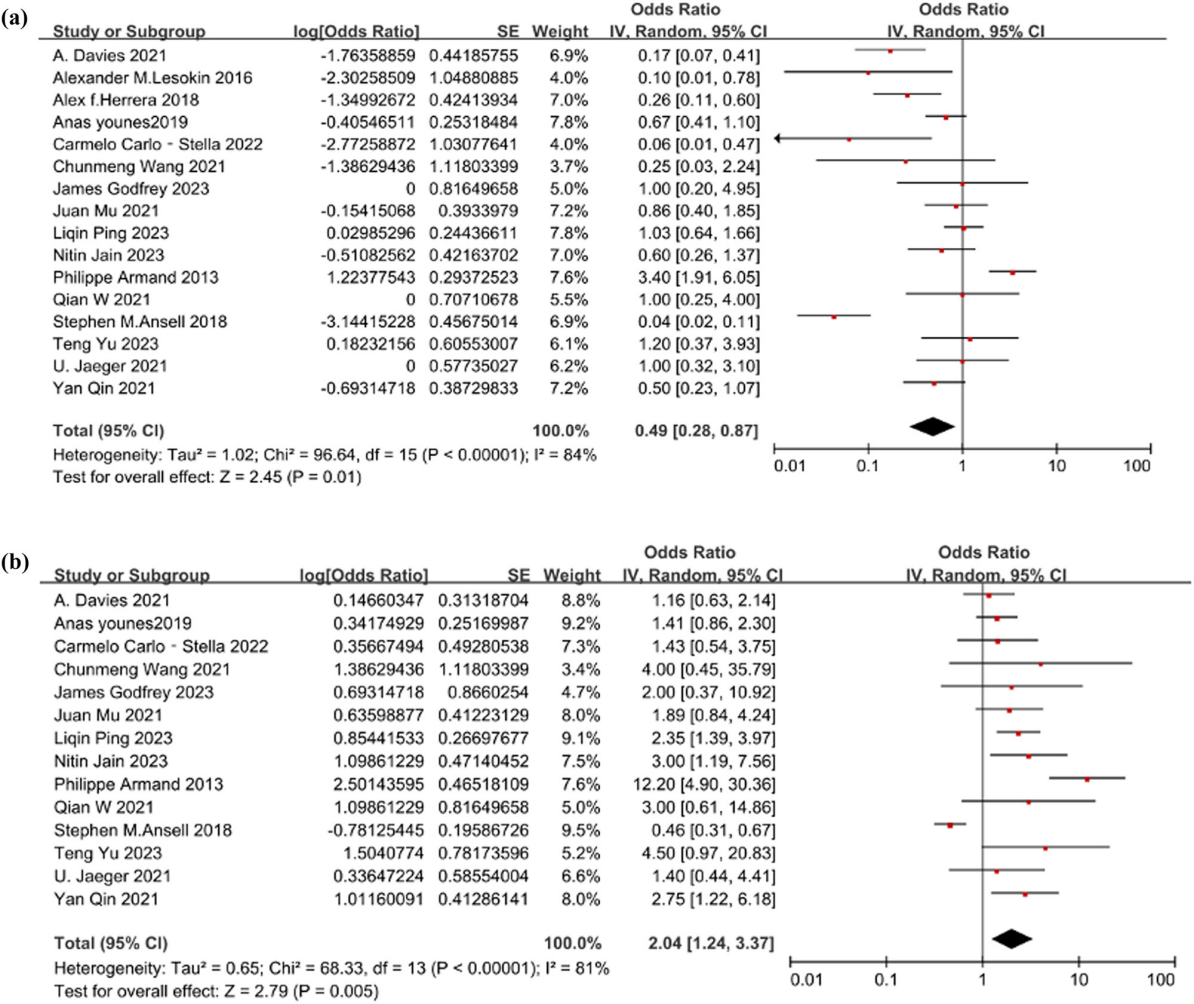
(a) The forest plot of the level of 1-year PFS. The heterogeneity test result was *Q* = 96.64 (*p* < 0.001) and *I* ^2^ = 84%. The estimated effect was [OR = 0.33, 95% CI 0.22–0.47; *p* = 0.01]. (b) The forest plot of the level of 1-year OS. The heterogeneity test result was *Q* = 68.33 (*p* < 0.001) and *I* ^2^ = 81%.The estimated effect was [OR = 0.67, 95% CI 0.55–0.77; *p* = 0.05].

The heterogeneity analysis for the level of PFS yielded *Q* = 96.64 (*p* < 0.001) and *I* ^2^ = 84%, indicating significant heterogeneity among the studies. Therefore, a random-effects model was employed for analysis. The estimated effect of PFS was [OR = 0.33, 95% CI 0.22–0.47; *p* = 0.01]. Figure 3a depicts the forest plot illustrating the level of PFS.

### 3.5 Heterogeneity test and estimated effect analysis of 1-year OS

The heterogeneity analysis for the level of OS yielded *Q* = 68.33 (*p* < 0.001) and *I* ^2^ = 81%, indicating significant heterogeneity among the studies. Therefore, a random-effects model was employed for analysis. The estimated effect of OS was [OR = 0.67, 95% CI 0.55–0.77; *p* = 0.05]. Figure 3b depicts the forest plot illustrating the level of OS.

### 3.6 Heterogeneity assessment and analysis of estimated effects on AEs

**Figure 4:**
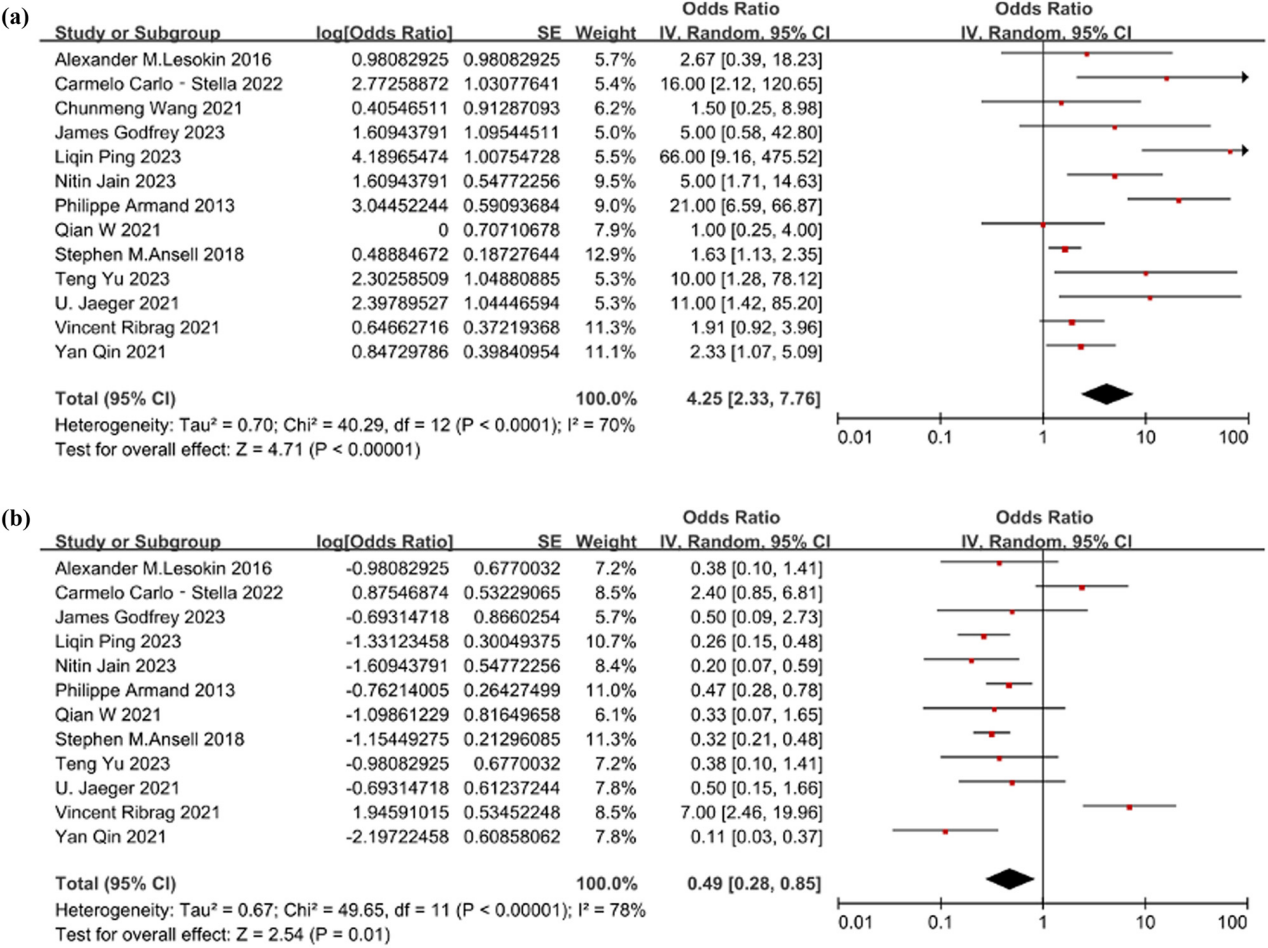
(a) The forest plot of the level of AEs. The heterogeneity test result was *Q* = 40.29 (*p* < 0.001) and *I* ^2^ = 70%. The estimated effect was [OR = 0.81, 95% CI 0.70–0.89; *p*＜0.001]. (b) The forest plot of the level of grade 3 AEs. The heterogeneity test result was *Q* = 49.65 (*p* < 0.001) and *I* ^2^ = 78%. The estimated effect was [OR = 0.33, 95% CI 0.22–0.46; *p* = 0.01].

The heterogeneity test revealed significant heterogeneity among studies for AEs of grades 1, 2 and 3, with *Q* = 40.29 (*p* < 0.001) and *I* ^2^ = 70%. Thus, a random-effects model was applied for analysis. The estimated OR for AEs was [OR = 0.81, 95% CI 0.70–0.89; *p* < 0.001], Figure 4a displays the forest plot illustrating AEs. Similarly, for grade 3 AEs, the heterogeneity test showed substantial heterogeneity, with *Q* = 49.65 (*p* < 0.001) and *I* ^2^ = 78%. Therefore, a random-effects model was employed. The estimated OR for grade 3 AEs was [OR = 0.33, 95% CI 0.22–0.46; *p* = 0.01]. Figure 4b depicts the forest plot of grade 3 AEs.

### 3.7 Subgroup analysis

**Figure 5:**
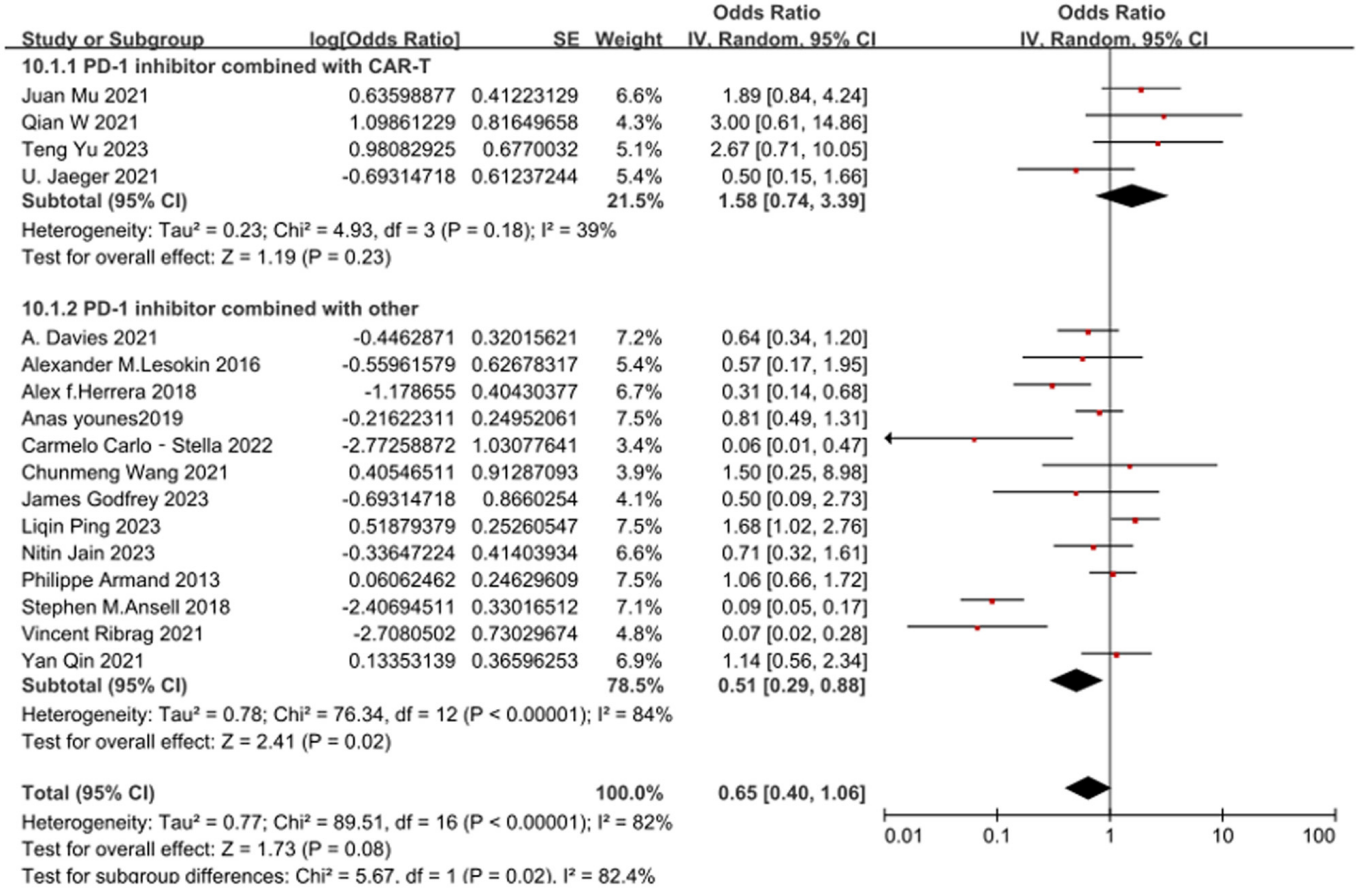
Subgroup analysis conducted based on whether CAR-T was combined.

Subgroup analysis was conducted based on whether CAR-T was combined, as illustrated in Figure 5. The overall heterogeneity across the included studies was significant, with *Q* = 89.51 (*p* < 0.001) and *I* ^2^ = 82%. This indicates substantial heterogeneity among the studies, necessitating the utilization of a random-effects model for analysis. Subgroup findings revealed that the PD-1 inhibitor combined with CAR-T group [OR = 0.61, 95% CI 0.43–0.77; *p* = 0.23], while the PD-1 inhibitor combined with other groups [OR = 0.34, 95% CI 0.22–0.47; *p* = 0.02]. The combination of PD-1 inhibitor with CAR-T demonstrated a significant improvement in ORR among R/R DLBCL patients.

### 3.8 Bias analysis

As depicted in Figure S1a and b respectively, the plots reveal that all data points are evenly distributed and symmetrical regarding the ORR and CRR. This symmetry suggests the absence of publication bias, thereby bolstering the credibility of the results. However, for the outcomes of 1-year PFS, 1-year OS, and AEs, as shown in Figures S2a, b, and S3, respectively, the symmetry among data points is notably poor. This discrepancy implies the presence of significant publication bias in these aspects.

## 4 Discussion

R/R DLBCL manifests as a biologically complex malignancy driven by multifactorial pathogenesis. Mounting evidence implicates viral triggers, dysregulated immune responses (both immunosuppressive states and hyperactive immunity), and environmental exposures as key etiological contributors [29]. Despite therapeutic advances, the prognosis remains dismal, with 5-year survival rates persisting below 30% in contemporary series. This persistent clinical challenge underscores the critical unmet need for rational development of novel combinatorial approaches and dynamic sequencing algorithms to overcome therapeutic resistance.

Contemporary oncology has witnessed immunotherapy revolutionize the therapeutic landscape through strategic immune system reprogramming. This paradigm harnesses host immunity to reinvigorate antitumor responses via three cardinal mechanisms: immune checkpoint modulation, T cell activation potentiation, and TME remodeling ultimately achieving durable tumor control [30,31]. At the molecular level, PD-1 and PD-L1, both immunoglobulin superfamily members, constitute co-inhibitory type I transmembrane proteins that orchestrate immune evasion. Functionally, PD-1 expressed on activated T lymphocytes engages PD-L1 expressed by malignant cells or stromal antigen-presenting cells, initiating inhibitory signaling cascades that attenuate T cell effector functions while establishing immune privileged niches for tumor progression [32].

At the molecular level, PD-1 activation on stimulated T lymphocytes initiates a sophisticated biochemical cascade: following ligand engagement, the receptor recruits SHP-1/SHP-2 phosphatases and downstream adaptor proteins, triggering catalytic dephosphorylation events that disrupt proximal signaling networks. This biochemical interplay culminates in three cardinal immunosuppressive effects: (1) blunted cytokine secretion (IFN-γ, TNF-α, IL-2); (2) proliferative arrest of antigen-specific T cell clones; and (3) breakdown of immune homeostasis through Treg/Th17 axis dysregulation collectively establishing a tumor permissive microenvironment [33,34]. These mechanistic insights have propelled monoclonal antibody based checkpoint blockade to the forefront of cancer immunotherapy, where PD-1/PD-L1 antagonists precisely intercept this co-inhibitory axis to restore immune-mediated tumor eradication.

Immunohistochemical profiling reveals distinct expression patterns of checkpoint molecules across lymphoma subtypes. PD-1 immunoreactivity is consistently detected in chronic lymphocytic leukemia/small lymphocytic lymphoma, follicular lymphoma, angioimmunoblastic T-cell lymphoma, and DLBCL [35]. Conversely, PD-L1 expression demarcates a separate pathological spectrum, being prevalent in Hodgkin lymphoma, anaplastic large cell lymphoma, and extranodal NK/T-cell lymphoma, while remaining undetectable in mantle cell lymphoma, marginal zone lymphoma, and Burkitt lymphoma [36]. Prognostically, multivariate analyses demonstrate a significant inverse correlation between PD-1 tumor infiltration density and survival outcomes in DLBCL (HR = 2.1, 95% CI 1.4–3.2; *p* < 0.01), whereas PD-L1 expression lacks comparable predictive value [37]. Intriguingly, the TME exhibits heightened PD-1+ cell infiltration in non-GCB DLBCL subsets characterized by CD30-/CD5-/EBER-phenotypes (72% vs 28%, *p* = 0.003), mirroring the striking survival disparity between GCB and non-GCB subtypes (5-year OS: 68% vs 41%, *p* < 0.001) [37]. Clinically, dual negative (PD-1−/PD-L1−) patients demonstrate superior treatment response rates (ORR: 84% vs 52%, *p* = 0.01) and 3-year survival (72% vs 35%, *p* < 0.001), a prognostic advantage mechanistically linked to the predominant PD-1+ phenotype in non-GCB biology [38].

PD-1/PD-L1 inhibitors can disrupt the interaction between PD-1 and its ligand PD-L1 on activated T cells, reversing T cell senescence and enhancing anti-tumor immune responses [7]. The meta-analysis results revealed an ORR of 0.40 (95% CI 0.29–0.51) and a CRR of 0.21 (95% CI 0.14–0.31) for PD-1/PD-L1 inhibitors in treating R/R DLBCL, consistent with the findings of Ding et al.’s study [39].

CAR-T cell therapy is a genetically engineered cellular treatment that offers a novel possibility for achieving long-lasting remission or even cure, acknowledged by the American Society of Clinical Oncology as the “2018 Advance” [40]. The first approved products include axicabtagene ciloleucel (axi-cel), tisagenlecleucel (tisa-cel), and lisocabtagenemaraleucel (liso-cel) [41–43], which function by programming autologous T cells to express CAR targeting the B-cell marker CD19.

Landmark phase 2 trials ZUMA-1, JULIET, and TRANSFORM demonstrated durable clinical benefit of CD19 directed CAR T-cell therapies in multiply R/R DLBCL [41]. Notably, the three FDA approved constructs (axi-cel, tisa-cel, liso-cel) achieved sustained remission in 30–40% of heavily pretreated patients (median 3 prior lines) over 24-month follow-up, including ASCT-refractory populations. However, emerging translational data elucidate TME-mediated resistance mechanisms particularly PD-L1 upregulation, Treg infiltration, and myeloid-derived suppressor cell accumulation that compromise CAR T-cell persistence and effector function *in vivo* [44,45].

Immunohistochemical analyses have consistently demonstrated pathological upregulation of PD-L1 in DLBCL TME, which correlates with adverse clinical outcomes including reduced PFS (median 8.2 vs 24.6 months, *p* < 0.001) and elevated relapse rates (HR = 3.4, 95% CI 2.1–5.5) [46]. Mechanistically, targeted disruption of the PD-1/PD-L1 axis serves as a dual-pronged immunotherapeutic strategy: not only potentiating host anti-lymphoma immunity through checkpoint reversal, but also enhancing CAR-T cell effector functions by mitigating terminal exhaustion phenotypes thereby synergistically improving therapeutic efficacy in B-cell malignancies [47,48].

Subgroup analysis indicated that the ORR of PD-1/PD-L1 inhibitors combined with CAR-T cell therapy was higher compared to monotherapy or combinations with other treatments, with rates of 0.61 and 0.34, respectively. Among the 17 studies analyzed, the phase 1b clinical trial by Qian et al. (NCT04381741) [24] reported the highest ORR and CRR following PD-1/PD-L1 inhibitor treatment. This trial involved eight patients aged 18–75 years with R/R DLBCL. Thirty days after modified T-cell infusion, the patients received six cycles of Tislelizumab (200 mg) every 3 weeks as an anti-PD-1 antibody.

Safety assessment revealed two patients (25%) developed grade ≥3 cytokine release syndrome (CRS) and two (25%) experienced grade 3 neurotoxicity, all managed effectively with protocol directed interventions. At 3-month follow-up (*n* = 7 evaluable), treatment responses stratified as five CR (71.4%), one PR (14.3%), and two progressive disease (28.6%), demonstrating enhanced therapeutic synergy between CAR-T and PD-(L)1 blockade in R/R DLBCL. This synergistic mechanism likely stems from CAR-T cells’ precision targeting capability selectively eliminating CD19+ malignant cells while preserving healthy tissues contrasted with conventional non-targeted therapies (e.g., chemotherapy) that indiscriminately damage proliferating cells. Despite these promising signals, current evidence remains limited by small cohort sizes (median *n* = 8 per study), necessitating validation through large-scale randomized controlled trials to establish clinical benefit risk profiles.

In terms of safety, research indicates that the incidence of fatal toxic reactions, such as myocarditis and pulmonary toxicity, associated with PD-1/PD-L1 inhibitors in lymphoma treatment is relatively low, with grade 3–4 AEs occurring in 1–14% of cases [49]. Overall, PD-1/PD-L1 inhibitors exhibit favorable tolerability among patients with R/R DLBCL. However, it is essential to note that while combination therapy may enhance clinical efficacy to some extent, it also brings about increased safety risks that warrant careful consideration. This is particularly relevant when approaching the threshold of drug efficacy, which may trigger severe autoimmune conditions and elevate the incidence of immune-related AEs [50].

This study reports an overall incidence rate of AEs at 0.81 (95% CI 0.70, 0.89), with a grade ≥3 AE incidence rate of 0.33 (95% CI 0.22, 0.46), aligning with the findings of Ding et al. [39]. None of the studies included in this analysis reported treatment-related deaths. The most prevalent AEs observed were fatigue, neutropenia, rash, nausea, diarrhea, and anemia, with anemia and neutropenia being the most common among the grade ≥3 AEs. Immune-related AEs, including rash, renal dysfunction, diarrhea, and hepatic dysfunction, were observed in only a small subset of patients.

The limitations of this study include: (1) the restricted pool of eligible studies (*n* = 17) with marked protocol heterogeneity – variability in therapeutic regimens and dosing schedules – resulted in substantial methodological heterogeneity (*I*² = 76.00%); (2) incorporation of two retrospective observational studies introduced baseline characteristic disparities and selection bias risks inherent to non-randomized designs; (3) critical survival endpoints (OS/PFS) were compromised by incomplete data granularity, precluding time-to-event meta-analysis via parametric survival models. These constraints collectively undermine the robustness of pooled effect estimates and limit generalizability of conclusions, necessitating cautious interpretation of therapeutic recommendations.

## 5 Conclusions

In summary, the meta-analysis of PD-1/PD-L1 inhibitors in the treatment of R/R DLBCL indicates limited therapeutic efficacy while demonstrating consistent safety profiles. Furthermore, the combination of PD-1/PD-L1 inhibitors with CAR-T cell therapy for R/R DLBCL yields satisfactory treatment outcomes. However, the lack of measurements for PD-1/PD-L1 expression levels in the TME and peripheral blood across the included studies precludes validation of their correlation with prognosis. Therefore, further research is warranted to elucidate this relationship.

## Supplementary Material

Supplementary material
