# Supplementary material for: Efficacy and safety of anti-PD-1/PD-L1 antibodies in patients with relapsed refractory diffuse large B-cell lymphoma: A meta-analysis
Source: Open Life Sci. 2025 Aug 5;20(1):20251129. doi: 10.1515/biol-2025-1129 (PMC12326303; doi:10.1515/biol-2025-1129)
Supplement: Supplementary material [file biol-2025-1129-sm.pdf]

Supplementary material

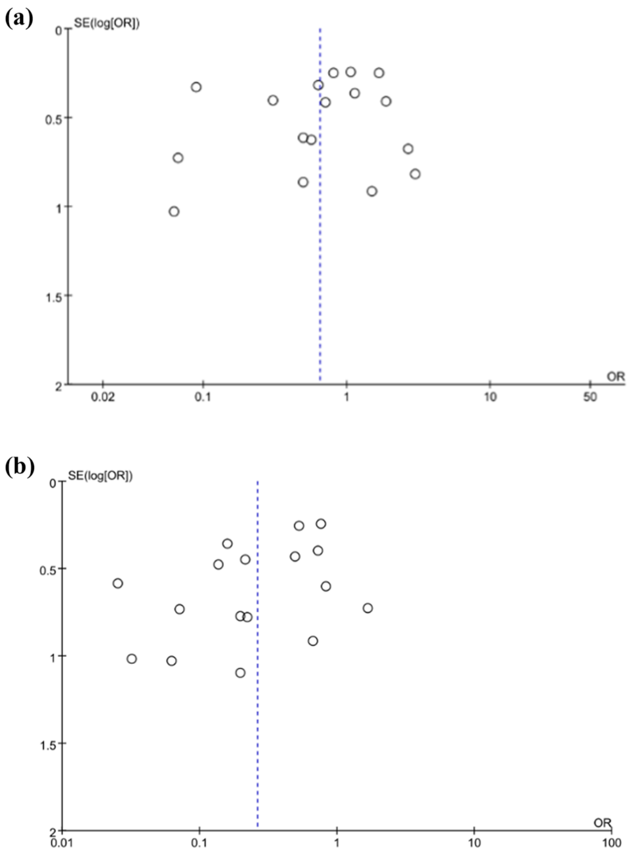

Figure S1: (a) The funnel plot of the level of ORR. The plot reveal that all data points are evenly distributed and symmetrical, (b). The funnel plot of the level of CRR. The plot reveal that all data points are evenly distributed and symmetrical.

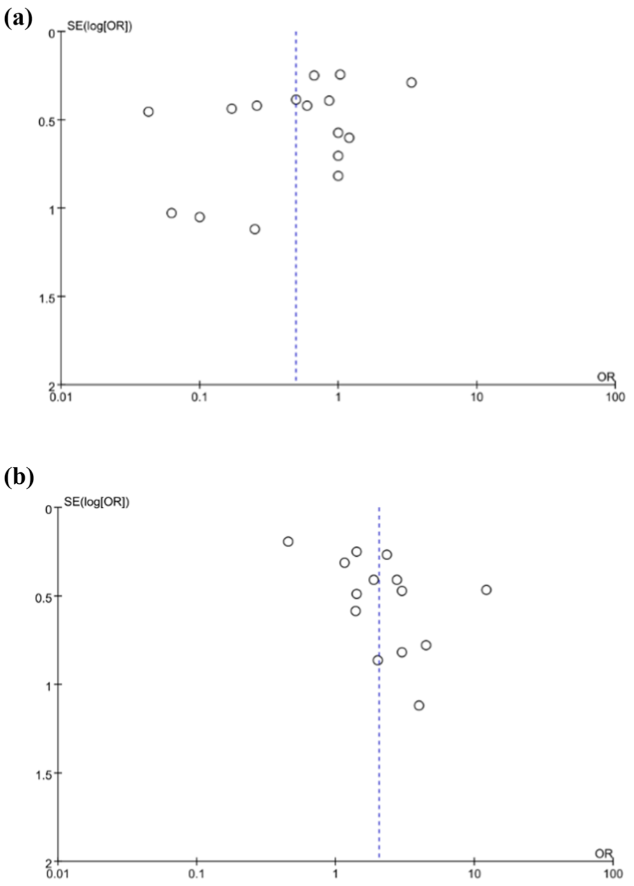

Figure S2: (a) The funnel plot of the level of 1-year PFS. The symmetry among data points is notably poor. (b) The funnel plot of the level of 1-year OS. The symmetry among data points is notably poor.

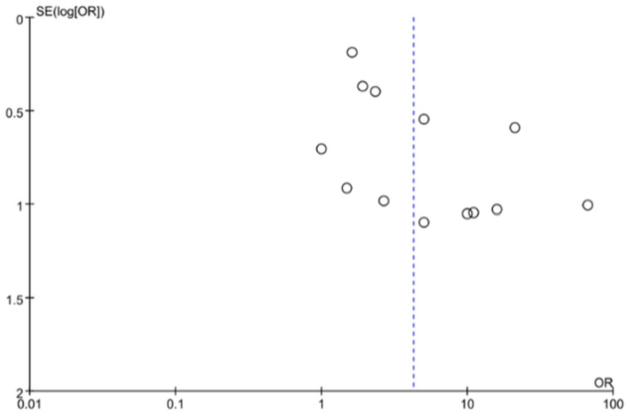

Figure S3: The funnel plot of the level of adverse events. The funnel plot of the level of adverse events. The symmetry among data points is notably poor.

Table S1: Study quality assessment based on the Minors

Author & year	A clearly stated aim	Inclusion of consecutive patients	Prospective collection of data	Endpoints appropriate to the aim of the study	Unbiased assessment of the study endpoint	Follow-up period appropriate to the aim of the study	Loss to follow up less than 5%	Prospective calculation of the study size
Philippe Armand 2013	2	2	1	2	0	2	2	0
Anas younes2019	2	2	2	2	0	2	2	0
Stephen M.Ansell 2018	2	2	1	2	2	2	2	0
Alexander M.Lesokin 2016	2	2	1	2	0	2	2	0
Vincent Ribrag 2021	2	2	2	2	0	0	2	0
Alex f.Herrera 2018	2	2	0	2	0	2	2	0
Liqin Ping 2023	2	2	1	2	2	2	2	0
Yan Qin 2021	2	2	1	2	2	0	2	0
A. Davies 2021	2	2	2	2	0	2	2	0
Juan Mu 2021	2	2	1	2	0	2	2	0
Teng Yu 2023	2	2	2	2	0	0	2	0
Chunmeng Wang 2021	2	2	2	2	0	2	2	0
Qian W 2021	2	2	0	2	0	2	2	0
James Godfrey 2023	2	2	1	2	0	2	2	0
Carmelo Carlo-Stella 2022	2	2	2	2	0	2	2	0
U. Jaeger 2021	2	2	2	2	0	0	2	0
Nitin Jain 2023	2	2	1	2	2	2	2	0
